# Nerve Growth

**Published:** 1919-01

**Authors:** 


***Nerve Growth.*** By Captain SYDNEY M. CONE, M. C., U. S. A. (From the Alder-Hey Military Orthopedic Hospital and the Thompson-Yates Pathological Laboratory, University of Liverpool).

Although the normal and most natural method of nerve growth after injury, which is commonly accepted, is by a sprouting from the central end, the author has been convinced that there is another method of nerve regeneration, sometimes called "autogenic". While in most instances nerves grow in direct continuity from a spinal connection, that does not always appear to be the case. In more than 450 specimens of war injured nerves, the study of nerve regeneration has been simplified by the neurokeration stain (see *British Journal of Surgery*, May 1918). The youngest, or "embryonal", nerve fibres are seen in their varicose shape and serpentine course in adhesions, intervening scar, central and peripheral ends of completely severed nerves, and become mature fibres on getting a spinal connection.

The bulbs at the central end of a divided nerve may contain nerves of different ages and sizes, but the peripheral end generally contains new-formed nerves of uniform size.

Nerves found in the distal (peripheral) ends of divided nerves are embryonal in most cases, and remain uniform in size and shape until connection is made with a centrally connected nerve trunk, when they mature.

Injured nerve ends are either firm and gray with punctiform, translucent areas well seen in pressure, or are softer, succulent and pinkish gray, resembling cellular granulation tissue. The first is made up of masses of 1-6 $\mu$ sized young nerves in interlacing fasciculi, with a matrix of vascular cellular connective tissue. The latter consists of a protoplasmic mass of cells, and young 1-3 $\mu$ sized nerves tending to form fasciculi.

When the report answered the latter description a few cases of early recovery (2-4 months) occurred. Where ends of the first type were recorded, very good results (4-8 months) were obtained. When the united ends show neither of these types as a predominant component, one may expect the usual functional results; i. e., in one to two years.

Clinical facts coupled with microscopic findings have convinced the author that early results are due to another method of nerve growth differing from the normal budding and downgrowth from the central end of the severed nerve.

In cases where the limb is used two to four months after suture, the material described as homogeneous is invariably found to be gray or pinkish gray—usually succulent or gelatinous.

The cases with well developed bundles of adult nerves took the normal time (8 months at least—usually longer) to recover function.

What is most commonly seen in the proximal (central) end, a few centimetres from the scar separating the cut ends, is a great proliferation of young varicose serpentine tendrils following vessels, or filling the old degenerated individual nerve fibres. Among these young nerve tendrils are innumerable elongated staff-shaped nuclei, and often there appears to be a transition of these end-to-end nuclei directly into nerve tendrils.

On examining the nerves in a case where the arm was amputated sixteen months after complete severance of the ulnar and musculo spiral, long rows of staff-shaped nuclei were discovered, abutting end to end. The ulnar peripheral end was bulbed and separated by three inches from the central cut end. The bulb consisted of both adult and young nerve fibres, with no formation of connective tissue from the nuclei.

It is the author's opinion that if a young embryonal nerve were invariably an ingrowth from surrounding nerves, one would more frequently see adult fibres among them. The young nerves are always surrounded by great numbers of elongated nuclei. These cells are neuroblasts. Guinea pig implants have been found to contain a great amount of keratinized, serpentine and varicose fibres, which are not located in the centre of the graft but 1/2 to 2 inches away. This leads to the conviction that the Selmann sheath cells may wander into surrounding tissues, and there carry on neuroblastic functions. Moreover, one has been taught to expect preliminary degenerative changes in nerves before regeneration takes place.

The independence of nerve growth is also proved by the finding of isolated nerve masses containing both young and adult nerves, degenerating and regenerating side by side in a matrix of cellular much nucleated material.

